# Exploring the Levels of Dental Anxiety in Greek Patients

**DOI:** 10.1016/j.identj.2025.04.006

**Published:** 2025-05-17

**Authors:** Metaxia Kritsidima, Sasha Scambler, Koula Asimakopoulou

**Affiliations:** a Faculty of Dentistry, Oral & Craniofacial Sciences, King's College London, London, UK; b Faculty of Health and Life Sciences, Oxford Brookes University, Oxford, UK

**Keywords:** Dental anxiety, Modified Dental Anxiety Scale, MDAS, Dental phobia, Greece

## Abstract

**Introduction:**

Dental anxiety is the apprehension experienced by an individual when confronted with matters related to dentistry, it impacts individuals' dental experiences and the dental healthcare providers’ professional lives, contributing to increased tension and potentially compromising performance. Levels of dental anxiety differ across cultures.

**Objective:**

To collect normative data on levels of dental anxiety in Greece from a representative sample of people attending their usual dentist for a routine dental appointment.

**Methods:**

A cross-sectional study was applied. 1313 Adults were recruited, as they attended 1 of 6 different dental settings and were scheduled for a routine dental appointment involving any of the following routine procedures: Check-up, hygiene, restoration, extraction, and pain relief. Their anxiety levels were assessed by applying the Modified Dental Anxiety Scale (MDAS), and by using the cut-off scores of 12 and 19 for assessing moderate and extreme anxiety, respectively.

**Results:**

32.6% per cent of the respondents were found to be dentally anxious, out of which 8.2 % experienced high levels of dental anxiety. The mean MDAS score for the total study population was 10.36 (SD= 4.639). The findings indicated that females and first-time dental patients reported significantly higher levels of dental anxiety than males and repeat attendees. However, dental anxiety levels did not vary significantly across different dental procedures, age groups and dental settings. These results are in line with those reported by White in the U.S. population, demonstrating comparable levels of dental anxiety across both populations.

**Clinical Significance:**

One in 3 patients visiting the dentist in Greece experience some level of dental anxiety. This finding underscores that dental anxiety is a prevalent public health concern in Greece. Dentists and healthcare providers should consider screening for dental anxiety that could lead to early identification and management.

## Introduction

Anxiety has been seen as an aversive psychological construct and has been defined as the feeling of apprehension experienced by an individual when confronted with matters that are dentally related.^1^ Dental anxiety is a common psychological condition^2^ characterized by fear, nervousness or stress related to dental treatment and visits to the dentist.^3^ Individuals with dental anxiety may experience high levels of fear and stress when faced with dental procedures,^4^ instruments,^5^ sounds,^6^ or the dental environment.^7^ Dental anxiety can range from mild to severe.^1^ It may be triggered by past negative experiences,^8^ the fear of pain,^4^ loss of control,^3^ or specific phobias like the fear of needles or anaesthesia.^9^

Dental anxiety impacts individuals' dental experiences and the professional lives of dental practitioners, contributing to increased tension and potentially compromising performance. Dealing with anxious patients can prolong visits, highlighting the challenge dental professionals face in managing dental anxiety.^10^^,^^11^ Dental anxiety can lead to avoidance of dental care, resulting in delayed treatment and potential oral health issues, and it interferes with the individual's life.^12^

Research into dental anxiety among Greek adult patients is limited. A randomized controlled trial (RCT) was conducted by our team and explored the effect of lavender scent on anticipatory anxiety in dental participants.^13^ Another study focused on validating the Greek version of the Modified Dental Anxiety Scale (MDAS).^14^ A third study,^14^ had significant limitations that undermined its credibility. Self-reporting measuring tools were implemented to assess dental anxiety; however, data was collected through interviews conducted by the dentist, which contradicts the self-reporting nature of the measuring tools and introduces potential bias. Additionally, administering questionnaires during dental surgery rather than in a neutral waiting area introduces a clear bias. Moreover, excluding individuals over 65 and first-time dental patients further diminishes the study's scope. Results were drawn by identifying the highly anxious patients using the MDAS measuring tool, and they assessed moderate anxiety levels using the SAS measurement, thus undermining the result’s credibility.

Studies on dental anxiety have demonstrated a correlation between levels of dental anxiety, age,^16^^,^^17^^,^^18^ gender,^19^^,^^20^^,^^21^ first-time visits,^16^^,^^20^^,^^22^^,^^23^ and the type of dental procedure to be performed.^4^^,^^24^ As no such literature exists for the Greek population, these aspects will also be explored.

Therefore, further exploration is required in the realm of dental anxiety research among Greek adults. This study aimed to determine the levels of dental anxiety in a sample of patients scheduled for a routine dental visit across several dental settings in Athens, Greece. A hypothesis will be tested to determine whether the levels of dental anxiety are equal to or less than 25,82%.^17^

## Methods

This cross-sectional study aimed to determine the prevalence of dental anxiety among patients attending dental clinics in Athens, Greece, by collecting normative data through surveys of individuals seeking routine dental appointments. The research included demographic data such as age group, gender, first dental visit, and the expected procedure such as dental check-up, cleaning, restoration, extraction and pain relief. Participants' dental anxiety levels were measured using the Modified Dental Anxiety Scale (MDAS) and by using the standard cut-offs of 12 and 19 for moderate and extreme anxiety, respectively.^14^^,^^25^^,^^26^

Ethical approval was granted from the Research Ethics Subcommittee of a UK university (reference: MRA-17/18-7620) and the Hellenic Data Protection Authority (HDPA, reference: α/α Αδείας: 1796, ΓΝ/Εξ/173/26-01-2017). The study targeted adults over 18 years old who were fluent in Greek. There was an ethical consideration to avoid postponing care for participants if they experienced significant pain during the study. Experiencing pain was not an exclusion criterion; those with scheduled regular dental appointments were invited to participate even if they were experiencing pain, which was acknowledged in the subsequent question about expected treatment as pain relief. Six dental clinics were randomly selected from diverse geographical locations in Athens and Piraeus.

Data collection materials included an introductory letter, a demographic information sheet, and the Greek version of the MDAS. Participants were informed about the study's purpose and assured of anonymity. The demographic sheet and MDAS were completed, with responses sealed in a designated envelope. Upon arrival for their scheduled dental appointment, while waiting for the treatment in the waiting room, the patients filled in the demographic sheet and MDAS questionnaire in a self-paced manner. These were handed to them by the secretary. All secretaries were blind to the study's purpose and design, and the treating dentist took no part in the procedure.

Data were analyzed using SPSS version 29, focusing on descriptive statistics, internal consistency via Cronbach’s alpha, and relationships between demographics and MDAS scores through Pearson correlation analysis and ANOVA. The researcher securely stored and managed all collected data.

## Results

**Fig. 1:**
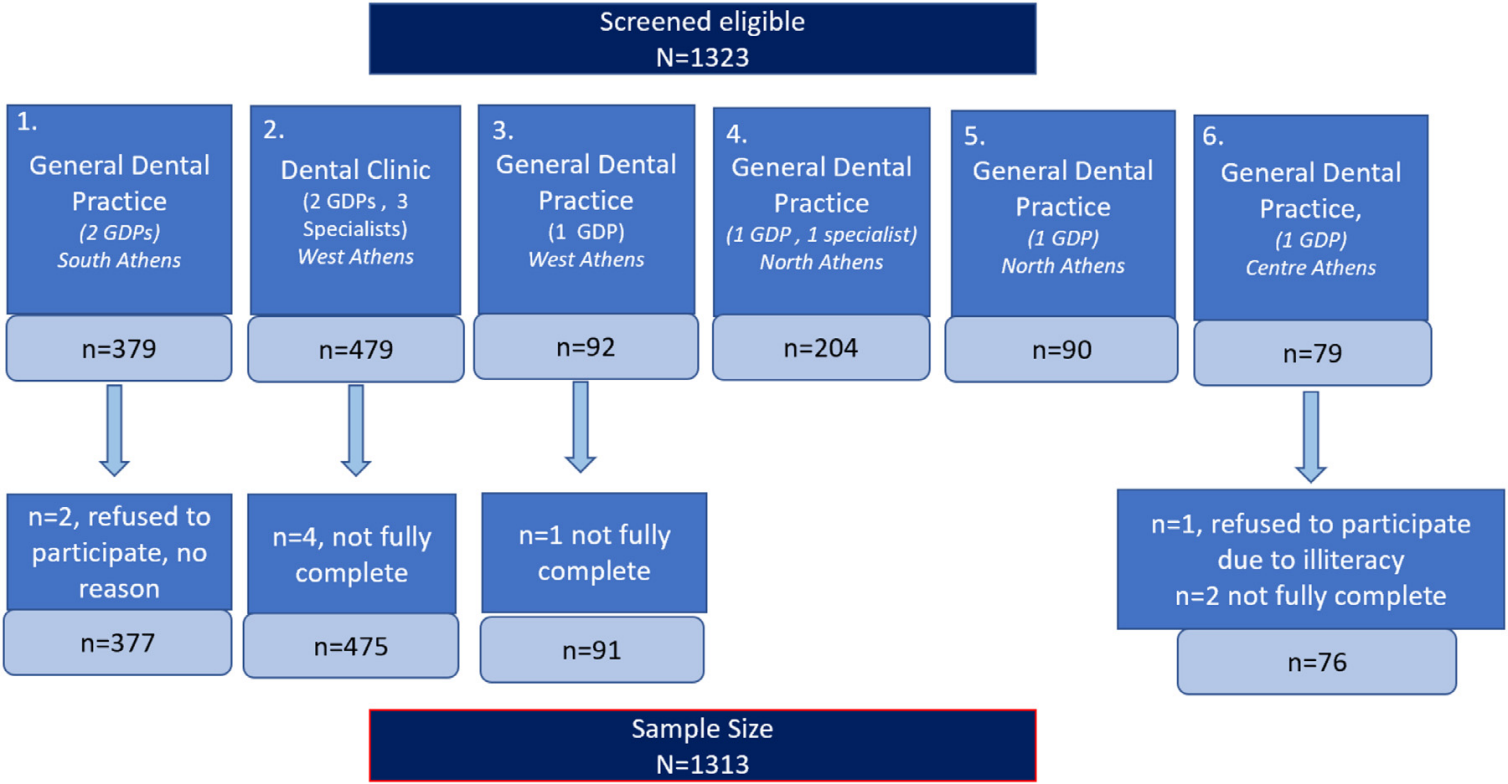
Flowchart of recruitment procedure to dental settings.

One thousand 3 hundred and twenty-three (N=1323) patients met the inclusion criteria. Seven submitted incomplete questionnaires, and 3 did not consent to participation. The response rate for this study was 99.24% (1313/1323). The flow chart below describes the allocation of participants to each dental setting (Figure 1).

Table 1 presents the demographic characteristics of the study participants, including age group, gender, first-time visit status, and the reason for their dental visit.

**Table 1:** Demographic characteristics.

		Number of participants (n)	0%	Accumulated Totals (N)
**Age group**	18-25	163	12%	163
	26-45	540	41%	703
	46-65	510	39%	1213
	Above 65	100	8%	1313
**Gender**	Male	509	39%	509
	Female	804	61%	1313
**First-time visit**	No	976	74%	976
	Yes	337	26%	1313
**Dental procedure to follow**	Check-up	464	35%	464
	Hygiene	441	34%	905
	Restoration	130	10%	1035
	Extraction	84	6%	1119
	Pain-relief	194	15%	1313

Data was grouped all together because no demographic differences existed between the practices’ patients.

**Fig. 2:**
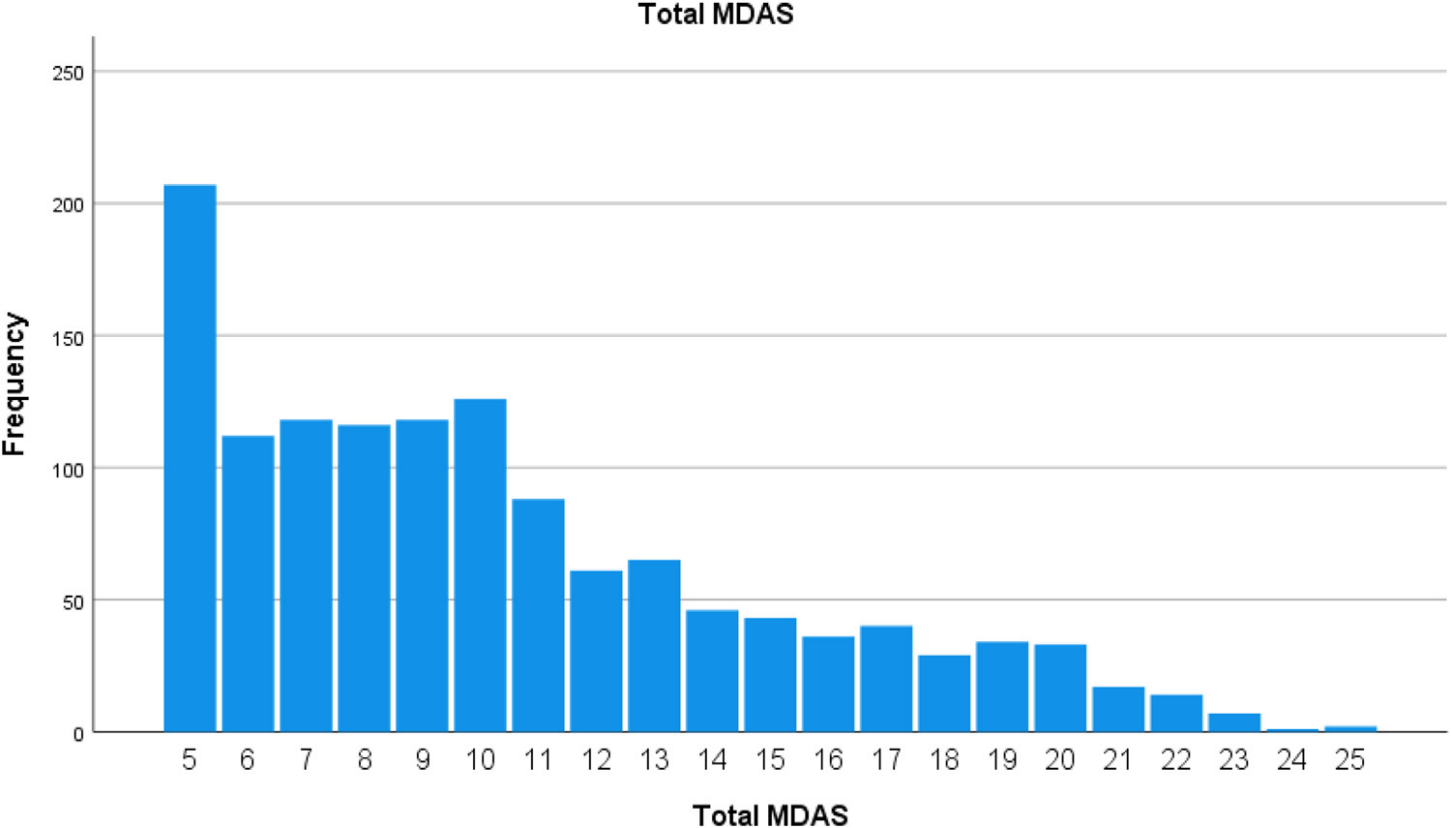
Total MDAS.

The mean MDAS score for the study population was 10,36 (SD = 4.639). Figure 2 is a graphic summary of the MDAS score distribution. The visual representation of the MDAS scores suggests anxiety levels to be skewed towards the lower end.

From the below table, females and younger individuals exhibited higher levels of anxiety. Patients who were undergoing a dental extraction or visiting the dental practice for the first time also demonstrated higher levels of anxiety. (Table 2) The implications of these findings will be thoroughly investigated.

**Table 2:** Mean MDAS Scores per Gender, Age group, First-time visit and Dental procedure.

Total MDAS				
		Mean	N	Std. Deviation
**Gender**	Female	10.87	804	4.741
	Male	9.55	509	4.358
**Age group**	18-25	10.74	163	4.549
	26-45	10.64	540	4.786
	46-65	10.06	510	4.553
	Above 65	9.70	100	4.303
**First-time visit**	No	10.07	976	4.461
	Yes	11.21	336	5.037
**Dental procedure to follow**	Check-up	9.98	464	4.587
	Hygiene	10.56	441	4.730
	Restoration	10.23	130	4.550
	Extraction	11.10	84	4.273
	Pain-relief	10.58	194	4.773

Participants were categorized into low, moderate, and high dental anxiety groups (Table 3)

**Table 3:** MDAS Scores of the N=1323 sample, based on MDAS cutoff points of 12 and 19.

MDAS Scores			
	Frequency	Percent	Cumulative Percent
**Low _(MDAS<12)_**	885	67.4	67.4
**Moderate _(MDAS=12-18)_**	320	22.4	91.8
**High _(MDAS≥19)_**	108	8.2	100
**Total**	1313	100	

MDAS demonstrated excellent internal consistency, with a Cronbach's alpha value of 0.85 (SEM = 0.08). This indicates that the scale's items are highly correlated and consistent across dental settings. The Kolmogorov-Smirnov test evaluated the skewness of the normality of the sample distribution for the MDAS-low scores. The test statistic was 0.431, indicating a significant deviation from normality (*P* < .01). Suggesting skewness as the sample distribution for the MDAS-low scores was not normally distributed.

Pearson chi-square tests were conducted for gender. Results show a statistically significant association between gender and dental anxiety (X² = 24.83, df = 2, *P < .*001). This suggests that women were significantly more likely to experience dental anxiety than men.

One-way ANOVAs were conducted to assess the association between dental anxiety levels and age groups, whether participants were attending their first dental appointment or whether the type of anticipated dental procedure influences dental anxiety levels. Results indicated a statistically significant difference in dental anxiety levels between first-time and repeat dental patients (F_(1323)_= 15.25, df = 1, *P = .*000), indicating that first-time dental patients had significantly higher dental anxiety levels than repeat patients. Results indicate no significant difference in dental anxiety levels and age groups (F_(1323)_= 2.222, df = 3, *P = .*084), indicating that there is no strong relationship between age and dental anxiety levels among participants of all ages. At the same time, results indicate no significant difference in dental anxiety levels among patients undergoing different dental procedures (F_(1323)_= 1.652, df = 4, *P = .*159). Although it appears as if anxiety fluctuates by procedure, these differences were not statistically significant.

One-way ANOVA was also performed to examine the association between dental anxiety levels and the type of dental setting. These results indicated no significant difference in dental anxiety levels among patients undergoing dental procedures in different settings (F_(1323)_= 1.651, df = 5, *P = .*144), suggesting that factors other than the type of setting may play a more important role in determining dental anxiety levels.

## Discussion

The findings of this study indicate that dental anxiety is a significant concern, affecting 32.6% of patients attending private dental practices in Greece, with a substantial 8.2% experiencing high levels of anxiety and 24,4 % experiencing moderate dental anxiety levels. This underscores that dental anxiety is a prevalent public health concern in Greece. That could prompt healthcare professionals and policymakers to consider strategies addressing dental anxiety.

These results align with those reported by White in the U.S. population,^17^ as their study design was similar to ours. In White and colleagues’ study, 25.82% of individuals visiting a dental practice were found to be dentally anxious, with 19% experiencing moderate to high anxiety and 6.82% indicating high dental anxiety. Their mean MDAS score was 10.19 (SD = 4.64), demonstrating comparable levels of dental anxiety across both populations.

Literature indicates significant variability in the prevalence rates of dental anxiety across different studies. This variation can be attributed mainly to methodological discrepancies, encompassing differences in assessment tools and the characteristics of study populations. For example, Ragnarsson reported that 28.4% of study participants in a population aged 25 to 74 experienced dental fear, with 5.1% exhibiting extensive dental fear with the Dental Anxiety Questionnaire (DAQ).^27^ Svensson and co-researchers, who also employed DAQ, reported 9.2% as highly anxious and 4.7% as extremely anxious within a population over 50 years old.^28^ Other studies to assess dental anxiety have employed the Dental Anxiety Scale (DAS), also showcasing a variation in findings, 12.4% as highly dentally anxious,^29^ 10.7%,^30^ and 16.6%.^31^ In the UK, Humprhris and colleagues assessed dental anxiety by measuring MDAS across the entire population,^16^ further underscoring the importance of methodological consistency in these evaluations. These variations highlight the significance of considering assessment tools, sample sizes, and the demographics of study populations when interpreting study results.

The present study investigated the prevalence of dental anxiety using the Modified Dental Anxiety Scale (MDAS) among patients attending private dental practices in Greece, aligning closely with similar study designs. This study assessed dental anxiety at a single point in time, contributing to a growing body of research on this topic. White and colleagues reported that among 308 dental patients, 19% exhibited moderate to high dental anxiety, while 6.82% displayed high dental anxiety, and MDAS was the measuring tool.^17^ In conclusion, the results of this study are pertinent to my objectives, as they provide valuable insights into the prevalence of dental anxiety in a specific demographic: patients attending a dental practice. Understanding these variations is crucial for interpreting findings and addressing dental anxiety effectively in diverse populations.

In this study in Athens, Greece, dental anxiety levels were examined among patients in various clinic settings. Normative data on anxiety levels were collected from individuals during routine dental appointments, and MDAS scores for anxiety measurement were recorded. The study focused on adults over 18 at 6 dental practices. Even though the prevalence of high dental anxiety (8.2%) within this study population is considered notably low, its impact on people, healthcare providers' experience, and the community is significant.

Anxiety related to dentistry is prevalent, with a third of the Greek study population exhibiting moderate to high levels of dental anxiety. Research indicates that forty-three per cent of individuals avoid dental appointments,^32^ and fifty-eight per cent of UK adults cite fear of the dentist as a partial reason for avoidance.^33^ Although this study was conducted exclusively within dental settings, it may have missed those with exceptionally high levels of dental anxiety due to avoidance behaviours. Dental anxiety impacts individuals' dental experiences and the professional lives of dental practitioners, contributing to increased tension and potentially compromising performance. Dealing with anxious patients can prolong visits, highlighting the challenge dental professionals face in managing dental anxiety.^10^^,^^11^

Additionally, cultural factors^34^ such as attitudes towards dental care,^35^ pain perception^36^ and communication styles^37^ can influence how dental anxiety is expressed.

Despite the differences mentioned earlier, studies on dental anxiety have consistently shown that dental anxiety is a prevalent problem worldwide. This consistency suggests that the general trend of elevated dental anxiety is robust and not merely an artefact of methodological differences. By tracking dental anxiety rates over time, researchers can identify emerging trends and assess the effectiveness of interventions aimed at reducing dental anxiety. Dental anxiety rates can also be compared across different cultures to understand how cultural factors influence the experience and expression of dental fear.

In this study, MDAS demonstrated, as expected, excellent internal consistency.^14^^,^^16^ However, the distribution appeared to be significantly skewed towards the low end for low anxiety, suggesting that either there is a significant cultural effect where people, when experiencing low anxiety levels, tend to report no anxiety at all or the measuring tool is not sensitive enough for low anxiety levels.

Other studies have described this skewness.^14^^,^^38^ Humphris and colleagues^38^ identified percentile rank accuracy as becoming less reliable for individuals who score low on the MDAS. They justified this finding by the “large number of the normative sample obtaining low scores” as they argued that when percentile ranks are based on a large group of individuals with similar scores, it becomes more difficult to pinpoint an individual's exact position within that group. In either case, it is undeniable that “this is not of much practical concern as there is little need for a precise estimate for low scores.”^38^ Additionally, this skewness does not influence the overall MDAS internal consistency, and the findings were concentrated on the moderate- and high-anxiety levels.

This cross-sectional study has demonstrated a high attendance rate (99%), which corroborates our previous study’s attendance rate of 99%^13^ and other Greek studies, 95%^14^ and 100%.^15^ This high level of attendance may suggest that Greek participants are engaged with the research topic; therefore, they perceive their input as valuable and important. Additionally, it may reflect positive perceptions, suggesting that the dentists or dental teams are well-regarded in Greece. This finding is also important as it indicates that MDAS is effective for use in a practice setting, as evidenced by the minimal number of refusals.

This study revealed that females exhibited significantly higher levels of dental anxiety compared to males. This finding aligns with previous research^14^^,^^38^^,^^39^^,^^40^ and suggests that gender may be a contributing factor to dental anxiety susceptibility.^18^^,^^21^ While other studies have also reported a difference between genders, these differences were not shown to be statistically significant.^14^^,^^16^ This diverse evidence may suggest that also other factors, such as gender role expectations or prevailing societal norms governing gender-appropriate actions and social gender behaviours norms, may have a significant impact on anxiety-associated behaviours.

First-time dental patients were found to have significantly higher anxiety levels than repeat patients, which agrees with the literature^14^^,^^16^^,^^22^^,^^23^ suggesting that the initial dental experience can have a profound impact on future dental anxiety. The anticipation and unfamiliarity associated with a first visit may contribute to heightened anxiety levels.

Younger individuals in this study showed elevated anxiety levels without significant association with high levels of dental anxiety. Research has shown mixed results regarding age and anxiety, with some studies agreeing with these findings and suggesting that younger individuals exhibit higher levels of anxiety,^16^^,^^17^ while others indicate a lower prevalence among younger adults.^19^^,^^41^ These inconsistent findings and lack of significant association between age groups and high levels of dental anxiety potentially highlight other factors indirectly associated with age; such factors can be limited dental exposure, a low level of dental environment familiarity, less shared dental information, or diverse past dental experiences.

Contrary to initial expectations,^4^^,^^25^ a significant difference in dental anxiety levels was not observed among patients undergoing different dental procedures. This suggests that the type of dental procedure may not be the primary determinant of anxiety levels and may suggest that other factors, such as individual predispositions, past experiences, and perceived pain, may play a more significant role.

The present study offers valuable insights into the prevalence and contributing factors of dental anxiety among patients attending dental settings in Greece. It is 1 of the first to provide normative data on dental anxiety specific to the Greek population. This highlights the importance of understanding how attitudes and beliefs about dental care can influence anxiety levels.

Although the results do not reflect the entire population, they accurately represent those seeking dental care. Given the high prevalence of dental anxiety in patients visiting the dentist, dentists and healthcare providers should be more proactive in identifying individuals who may be experiencing anxiety related to dental treatment. This could involve routine screening for anxiety during initial dental examinations or providing information about the availability of anxiety assessment tools. Practice screening for dental anxiety could lead to early identification and management. Future studies should address the current research's limitations by assessing the prevalence of dental anxiety in the whole Greek population.

## Conclusion

Based on the insights gained from this study, it is clear that dental anxiety is a prevalent issue among patients in Greece, influenced significantly by factors such as gender and being a first-time visitor. To effectively address this challenge, targeted interventions and personalized care strategies should be developed, emphasizing the need for routine anxiety screenings during initial consultations. Furthermore, understanding individual patient experiences and perceptions of the dental environment is crucial in tailoring anxiety management approaches. While this study forms a foundation for further research, it also underscores the need for broader investigations into the influencing factors of dental anxiety across the whole population, which can ultimately enhance dental practices and improve patient outcomes.

## Conflict of interest interest

The authors declare the following financial interests/personal relationships which may be considered as potential competing interests:

Metaxia Kritsidima reports statistical analysis and writing assistance were provided by King’s College London. If there are other authors, they declare that they have no known competing financial interests or personal relationships that could have appeared to influence the work reported in this paper.

## References

[1] Freeman R., Humphris G. (2006). http://library.dundee.ac.uk/F/?func=direct&local_base=DUN01&doc_number=000543259.

[2] Silveira E.R., Cademartori M.G., Schuch H.S., Armfield J.A., Demarco F.F. (2021). Estimated prevalence of dental fear in adults: a systematic review and meta-analysis. J Dent.

[3] Appukuttan D.P. (2016). Strategies to manage patients with dental anxiety and dental phobia: literature review. *C* Alves *ical, cosmetic and investigational dentistry*.

[4] Lin C.S., Wu S.Y., Yi C.A. (2017). Association between Anxiety and Pain in Dental Treatment: A Systematic Review and Meta-analysis. J Dent Res.

[5] Atram Abdulrahman A.Al, Singh Smita, Bhardwaj Atul, Fadalah Mousa Khalid Abu (2016). Evaluation of fear and anxiety associated with instruments and treatment among dental patients. International Journal of Contemporary Medical Research.

[6] Antoniadou M., Tziovara P., Antoniadou C. (2022). The effect of sound in the dental office: practices and recommendations for quality assurance-a narrative review. Dent J (Basel).

[7] Yap A.U., Kwan Y.Y., Kok L., Lee X.F., Lee D.Z.R. (2022). Dental environment and practitioner preferences of southeast Asian youths with dental fear/anxiety. Int J Dent Hyg.

[8] Weiner B.K. (2007). Difficult medical problems: on explanatory models and a pragmatic alternative. Med Hypotheses.

[9] Armfield J.M., Heaton L.J. (2013). Management of fear and anxiety in the dental clinic: a review. Aust Dent J.

[10] Humphris G., Ling MS. (2000).

[11] Kent G.G., Blinkhorn A.S. (1991). The psychology of dental care.

[12] Hoffmann B., Erwood K., Ncomanzi S., Fischer V., O'Brien D., Lee A. (2022). Management strategies for adult patients with dental anxiety in the dental clinic: a systematic review. Aust Dent J.

[13] Kritsidima M., Newton T., Asimakopoulou K. (2010). The effects of lavender scent on dental patient anxiety levels: a cluster randomised-controlled trial. Community Dent Oral Epidemiol.

[14] Coolidge T., Arapostathis K.N., Emmanouil D. (2008). Psychometric properties of Greek versions of the Modified Corah Dental Anxiety Scale (MDAS) and the Dental Fear Survey (DFS). BMC Oral Health.

[15] Makri C., Alexias G., Togas C., Chasiotis V. (2021). Evaluation of dental anxiety and of its determinants in a Greek sample. Int J Caring Sci.

[16] Humphris G.M., Dyer T.A., Robinson P.G. (2009). The modified dental anxiety scale: UK general public population norms in 2008 with further psychometrics and effects of age. BMC Oral Health.

[17] White A.M., Giblin L., Boyd L.D. (2017). The Prevalence of Dental Anxiety in Dental Practice Settings. J Dent Hygiene: JDH.

[18] Feingold A. (1994). Gender differences in personality: A meta-analysis. Psychol Bull.

[19] Saba Z., Katirci G. (2023). Relationship between dental anxiety levels and oral health among dental patients in Turkey: a cross-sectional study. BMC Oral Health.

[20] Coolidge A. (1994). Gender differences in personality: a meta-analysis. Psychol Bull.

[21] Kirkpatrick D.R. (1984). Age, gender and patterns of common intense fears among adults. Behav Res Ther.

[22] Winkler C.H., Bjelopavlovic M., Lehmann K.M., Petrowski K., Irmscher L., Berth H. (2023). Impact of dental anxiety on dental care routine and oral-health-related quality of life in a german adult population-a cross-sectional study. J Clin Med.

[23] Saheer A., Majid S.A., Raajendran J., Chithra P., Chandran T., Mathew R.A. (2022). Effect of dental anxiety on oral health among the first-time dental visitors: a hospital-based study. J Pharm Bioallied Sci.

[24] Yakar B., Kaygusuz T.Ö., Pırınçcı E. (2019). Evaluation of dental anxiety and fear in patients who admitted to the faculty of dentistry: which patients are more risky in terms of dental anxiety. Ethiop J Health Sci.

[25] Humphris G., Morrison T., Lindsay S.J.E. (1995). The Modified Dental Anxiety Scale: UK norms and evidence for validity. Commun Dent Health.

[26] Corah N.L., Gale E.N., Illig S.J. (1978). Assessment of a dental anxiety scale. J Am Dent Assoc.

[27] Ragnarsson E. (1998). Dental fear and anxiety in an adult Icelandic population. Acta Odontol Scand.

[28] Svensson L., Hakeberg M., Boman U.W. (2016). Dental anxiety, concomitant factors and change in prevalence over 50 years. Commun Dent Health.

[29] Bell R.A., Arcury T.A., Anderson A.M. (2012). Dental anxiety and oral health outcomes among rural older adults. J Public Health Dent.

[30] Liddell A., Locker D. (1997). Gender and age differences in attitudes to dental pain and dental control. Community Dent Oral Epidemiol.

[31] Schuller A.A., Willumsen T., Holst D. (2003). Are there differences in oral health and oral health behavior between individuals with high and low dental fear?. Community Dent Oral Epidemiol.

[32] Todd J.E., Lader D., Great Britain, University of Birmingham, University of Newcastle upon Tyne (1991).

[33] Todd J.E., Walker A., Dodd P. (1982).

[34] Folayan M.O., Idehen E.E., Ojo O.O. (2004). The modulating effect of culture on the expression of dental anxiety in children: a literature review. Int J Paediatr Dent.

[35] Smith A., MacEntee M.I., Beattie B.L. (2013). The influence of culture on the oral health-related beliefs and behaviours of elderly chinese immigrants: a meta-synthesis of the literature. J Cross Cult Gerontol.

[36] Dawson A., List T. (2009). Comparison of pain thresholds and pain tolerance levels between Middle Easterners and Swedes and between genders. J Oral Rehabil.

[37] Yuan S., Freeman R., Hill K., Newton T., Humphris G. (2020). Communication, trust and dental anxiety: a person-centred approach for dental attendance behaviours. Dent J (Basel).

[38] Humphris G., Crawford J.R., Hill K., Gilbert A., Freeman R. (2013). UK population norms for the modified dental anxiety scale with percentile calculator: Adult dental health survey 2009. BMC Oral Health.

[39] King K., Humphris G.M. (2007). Evidence to confirm the cut-off for screening dental phobia using the Modified Dental Anxiety Scale. Soc Sci Dent.

[40] Armfield J.M., Spencer A.J., Stewart J.F. (2006). Dental fear in Australia: Who's afraid of the dentist?. Aust Dent J.

[41] Thomson W.M., Stewart J.F., Carter K.D., Spencer A.J. (1996). Dental anxiety among Australians. Int Dent J.

